# COVID-19 Vaccination Acceptance Among the Diabetic Population in the Northwestern Region of Romania: Insights From an Autofill Survey

**DOI:** 10.7759/cureus.81464

**Published:** 2025-03-30

**Authors:** Alecsandra Andreea Budihoi, Bogdana Adriana Nasui, Nina Ciuciuc, Alexandra-Ioana Rosioara, Oana Uzarciuc-Coldea, Anamaria Apan, Tudor Calinici, Valeria Pop, Monica Popa

**Affiliations:** 1 Department of Infectious Diseases and Epidemiology, Iuliu Hațieganu University of Medicine and Pharmacy, Cluj-Napoca, ROU; 2 Department of Community Medicine, Research Center in Preventive Medicine, Health Promotion and Sustainable Development, Iuliu Hațieganu University of Medicine and Pharmacy, Cluj-Napoca, ROU; 3 Nutrition, Nutri Diagnostic Medical Office, Cluj-Napoca, ROU; 4 Department of Pharmacology, Physiology, and Pathophysiology, Faculty of Pharmacy, Iuliu Hațieganu University of Medicine and Pharmacy, Cluj-Napoca, ROU; 5 Department of Medical Informatics and Biostatistics, UMF Cluj-Napoca, Cluj-Napoca, ROU; 6 Faculty of Environmental Science and Engineering, Babeş-Bolyai University of Cluj-Napoca, Cluj-Napoca, ROU

**Keywords:** acceptance, covid-19, diabetes, high-risk population, northwest region, questionnaire, romania, vaccination

## Abstract

Introduction

The global experience of COVID-19 has highlighted the underestimated importance of vaccination as a preventive measure. Vaccine acceptance can be influenced by multiple factors, which can be significantly reduced through improved vaccination promotion strategies. This study aims to identify these factors and explore potential strategies to enhance vaccine uptake among the diabetic population.

Materials and methods

A cross-sectional study was conducted on 189 patients diagnosed with type 1 or 2 diabetes. The selected patients were from the northwestern region of Romania, the largest and most significant area for diabetes treatment. We used an adapted, pretested, self-administered questionnaire developed by the authors in collaboration with other medical professionals. Participants completed a 27-item survey covering personal sociodemographics, medical history related to diabetes, comorbidities and COVID-19, level of education, religion, ethnicity, type of COVID-19 vaccine administered, and attitude toward COVID-19 vaccination, including acceptance and hesitancy. Descriptive and inferential statistics were performed, with results presented as percentages and associations. The difference between groups that accepted or refused vaccination was examined using the Chi-square test, with a p-value < 0.05 considered statistically significant. If the results were statistically significant, the odds ratio (OR) was calculated with a 95% confidence interval.

Results

The participants were 96 (50.8%) females and 93 (49.2%) males. Most patients, 116 (61.4%), were between 51 and 70 years old. Moreover, 56 (29.6%) had only completed high school in terms of education level. From the medical history, 186 (98.4%) had type 2 diabetes, 162 (85.7%) were taking oral antidiabetics, 94 (49.7%) had cardiovascular diseases, and 161 (85.2%) had at least one COVID-19 infection. One hundred seventy-three patients were vaccinated against COVID-19. The most common vaccine, 143 (82.7%), was Pfizer. The principal determinant for vaccine acceptance, identified by 109 (63%) participants, was individual health and the well-being of others. For those who did not choose to get vaccinated, fear of side effects (7; 43.8%) was the main reason. Regarding the administration of a third dose, the main reason for vaccine hesitancy is overcome by personal perception (22; 20.6%), while that for vaccine acceptance remains the same, i.e., individual health and the well-being of others (56; 84.8%). Vaccine acceptance had a statistically significant relationship with variables like marital status, age, orthodox religion, ethnicity, occupation, and education.

Conclusions

Variables such as marital status, religion, and age can positively influence vaccination uptake. Comprehensive education on preventive medicine, starting from an early age and integrated within the healthcare system, is essential for fostering understanding and acceptance of vaccination.

## Introduction

Although vaccines are a cornerstone of modern medicine and public health, vaccine hesitancy remains a significant risk factor contributing to global non-vaccination. It is defined as a possible psychological state of indecisiveness when it is necessary to apply the vaccination [1]. This hesitation poses a public health challenge, as it can lead to increased morbidity and mortality, with vulnerable groups, such as diabetic patients, being at the highest risk.

In 2019, the World Health Organization (WHO) listed vaccine hesitancy among the top 10 global health threats [2]. The novel severe acute respiratory syndrome coronavirus (SARS-CoV-2) caused an important respiratory syndrome, coronavirus disease 2019 (COVID-19), which spread rapidly worldwide. Despite a considerable number of COVID-19-related worldwide deaths (7,052,472 on June 23, 2024, declared by the WHO), the benefits of vaccination have not been universally acknowledged, and many people remain hesitant to get vaccinated [3]. Over the years, numerous lives have been lost, and disbelief has been one of the factors contributing to the high mortality rates [4]. It is important to mention that vaccines eradicated smallpox, one of the most transmissible diseases with severe complications, within five years after the introduction of EPI (Expanded Program on Immunization) [5].

A survey of COVID-19 vaccine acceptance across 23 countries showed an increase from 75.2% in 2021 to 79.1% in 2022. By contrast, vaccine hesitancy increased in eight countries and was most related to misrepresentation and misinformation [6].

A possible cause that may dampen vaccination acceptance is the misinterpretation of the aggressiveness of the new SARS-CoV-2 variants. As the new virus variants are less aggressive, the public may use this as a reason for not getting vaccinated [7]. New research data show that some vaccines have reduced efficacy and effectiveness against COVID-19 caused by the beta variant (B.1.351) and the delta variant (B.1.617.2) [8]. The general population can also use this as a reason for vaccine hesitancy.

Promoting the importance of vaccination is the best way to achieve high levels of individual protection and sufficient herd immunity to successfully control vaccine-preventable diseases and sustain a high percentage of vaccinal coverage. According to health organizations, each country has a recommended vaccination coverage rate. However, the vaccination coverage rate (VCR) remains below these recommended levels [9,10]. 

On December 8, 2023, the National Centre for Surveillance and Control of Communicable Diseases (CNSCBT) in Romania released a summary regarding the vaccination coverage rate for children born in July 2021, aged 18 months. The vaccination coverage rate did not exceed the desired target of 95%. The main reasons for non-vaccination are not showing up to the doctor, being born or living in another country, and medical contraindications [10].

Romania has reported numerous cases of the flu over the past two years. A study from Romania showed a low vaccination rate against flu and COVID-19 in 2022-2023. They found a strong association between being vaccinated for flu and COVID-19 and being a healthcare worker (HCW). The area of residence was a factor for COVID-19 vaccination; people from urban areas have been immunized in greater numbers. No association between age, gender, and COVID-19 was found [11]. Regarding Romania, vaccine acceptance among special populations has not been extensively studied and remains underreported [12].

During the pandemic, Romania had one of the lowest vaccination rates against COVID-19. As highlighted in a study, this low vaccination rate is linked to several factors, including deficiencies in the medical and socioeconomic sectors. Some factors are related to political issues, misinformation of the population by anti-vaccine movements, or the low support from the Romanian Orthodox Church (ROC) [13].

The need for vaccination among individuals with diabetes is higher, as they belong to a high-risk category for developing severe complications if infected with COVID-19. Patients with diabetes are informed about the negative consequences an infection can have on their health if preventive measures are not taken. Nonetheless, some of them decide not to get vaccinated. The most common barriers to vaccine acceptance include misinformation, lack of information, mistrust, health concerns, and external influences [14].

It is known that viral infections can affect glycemic levels. During the COVID-19 pandemic, the hospitalization rate for diabetic patients was notably high. Diabetic patients have a higher mortality rate and a shorter survival time than non-diabetic patients and are more predisposed to severe metabolic complications [15]. A study conducted among hospitalized COVID-19 patients in England found that 18.3% had type 2 diabetes, and 26.4% of diabetic patients died during the survey period (adjusted hazard ratio 1.23, 95% confidence interval (CI) 1.14-1.32) [16].

The northwestern region of Romania is a reference area for patients with diabetes. Many individuals with diabetes come to the hospital for evaluation and treatment. This region hosts clinics and specialized centers that address the specific needs of diabetic patients [17].

The study aims to see if demographic, behavioral, and medical characteristics of patients with diabetes in the northwestern region of Romania can influence their COVID-19 vaccine acceptance since vaccine hesitancy may pose a significant risk to their health. Through this study, we highlight the possible factors that can be involved in COVID-19 vaccine acceptance within a vulnerable category: patients with diabetes mellitus. Understanding these factors allows us to reduce potential complications or prevent possible deaths.

## Materials and methods

Study area

Cluj-Napoca, located in northwestern Romania, is the largest and most populous city, serving as the administrative center of Cluj-Napoca. As of 2024, the city is home to approximately 334,387 residents. Cluj-Napoca is well-regarded for its advanced healthcare services, particularly in the management of chronic conditions, such as diabetes, with many patients seeking treatment here due to the city's established medical infrastructure.

**Figure 1:**
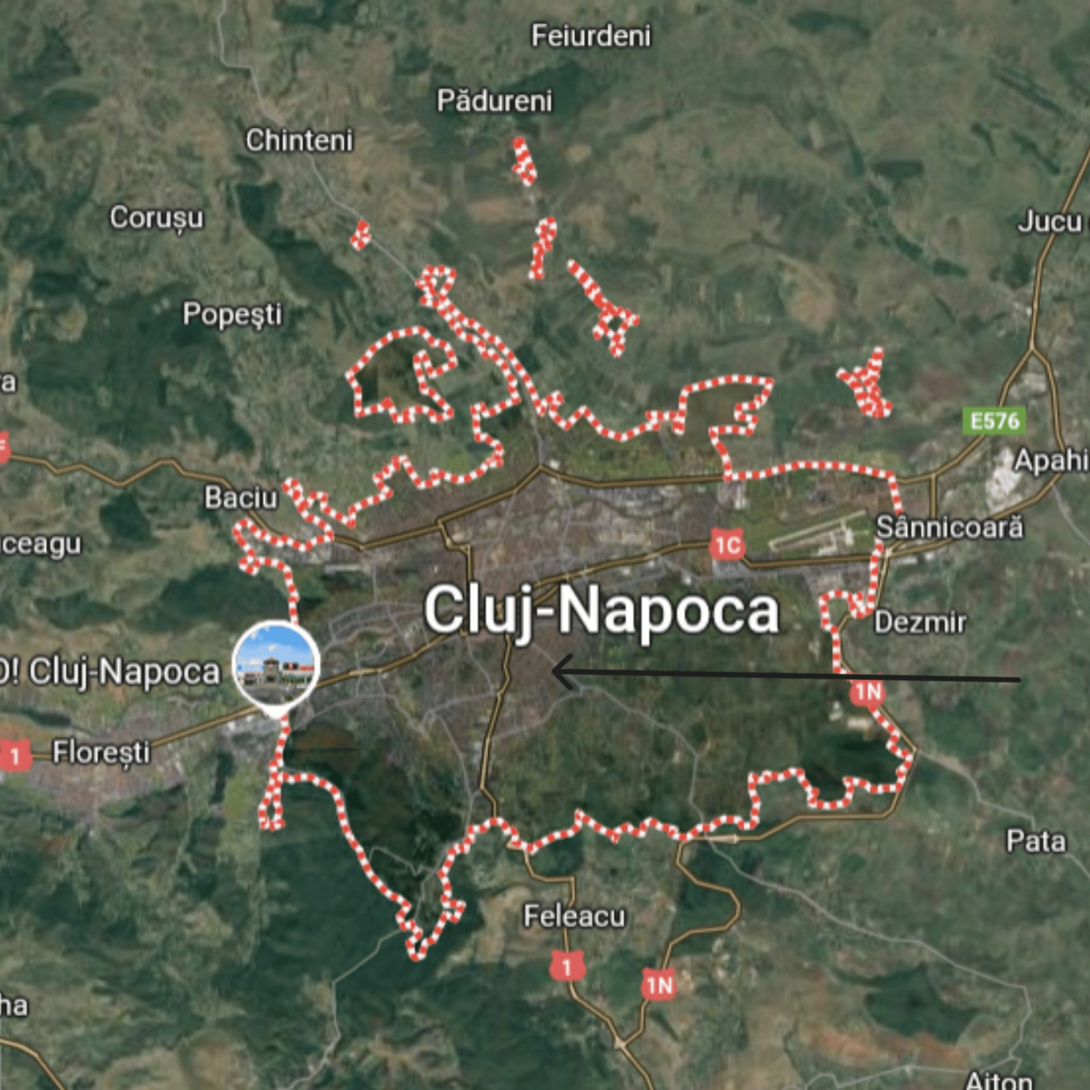
Living area of the responders (n =189) of the survey on the acceptance of COVID-19 vaccination in Romania (Cluj-Napoca County) Image reproduced with permission from Google Maps, 2025.

Figure 1 shows the living area of the responders (n =189) of the survey on the acceptance of COVID-19 vaccination in Romania (Cluj-Napoca County).

Study design and participants

We conducted a cross-sectional study on patients diagnosed with diabetes in two medical clinics, the Podiatry Clinic and the Nutridiagnost Medical Center, from Cluj County, the largest and most important medical area in northwestern Romania. Using the OpenEpi program, we estimated a minimum sample size of 135 participants. The response rate used for sample size estimation was based on the local context of the study, which focused on diabetic patients in the northwestern region of Romania. Given the favorable demographic, educational, and socioeconomic factors of patients in this region, as well as their higher trust in healthcare services and accessibility to treatment centers, participation rates are expected to be more favorable. This figure was deemed appropriate to ensure statistical reliability and the feasibility of the study while adhering to resource constraints.

A total of 250 patients were approached and consented to participate in the study, but only 200 met the eligibility criteria, and 189 of them agreed to participate in the research. The inclusion criteria are patients diagnosed with diabetes, between 18 and 90 years old. To reduce potential study risks and possible confounding variables that could affect the outcome of interest, we excluded patients with cognitive impairment and patients who declined participation in the research. Patients with cognitive impairment were excluded to ensure the accuracy and reliability of survey responses, as these individuals may have difficulty understanding the questions or providing informed answers. Following ethical research guidelines, patients who declined participation were excluded to respect their autonomy and voluntary consent.

Instrument of evaluation

For this research, the authors developed the questionnaire in collaboration with other medical professionals, drawing upon preexisting questionnaires and insights from the specialized literature. The questionnaire used in this study is adapted from existing sources, with modifications to align with this research [18,19]. The English and Romanian version of the Vaccination Acceptance Questionnaire is provided in Appendix A.

The questionnaire was pretested on a small, representative sample of 25 diabetic patients from the target population, which was not part of the actual study sample. This pretest sample was carefully selected to reflect the demographic and medical characteristics of the intended participants, including factors such as age, gender, type of diabetes, and other relevant medical conditions. Using a sample that mirrors the main study population, the pretest helped identify potential issues with the questionnaire's clarity, question relevance, and overall structure, ensuring that the final survey would be well-suited to collect reliable data from the participants. Following the pretest, necessary adjustments were made to enhance the clarity and reliability of the final questionnaire. The responses from the pretested sample were not included in the study analysis and results. The time required to fill in the questionnaire was approximately 25-35 minutes. 

To collect our data, we distributed a questionnaire between January 2023 and June 2024 among patients with type 1 and type 2 diabetes. The patients were referred to two medical clinics in northwestern Romania, the largest and most important region of the country. Participants self-administered the questionnaire in the waiting room, one of the available spaces for this process. Before the questionnaire was administered, written informed consent was obtained from each participant in the study. We used 27 items: 25 questions with one or multiple-choice and two open-ended questions.

The questionnaire included multiple sections addressing key variables relevant to the study. The first section covered sociodemographic and general characteristics, including age, gender, marital status, and religion.

The second section assessed occupational status, categorized as retired, unemployed, or employed in various fields. Educational level was classified into primary school, high school, trade school, or college. The next section focused on diabetes-related characteristics, such as diabetes type (type 1 or type 2), insulin treatment (yes/no), the number of daily doses (1, 2, 3, or 4), and the type of insulin used. In addition, the participants were asked whether they were undergoing oral antidiabetic treatment (yes/no) and to specify the name of the medication if applicable. The questionnaire also evaluated the presence of diabetes-related complications, including eye damage, kidney disease, and heart disease. Diabetic foot, skin problems, or no complications.

Participants were further asked about their COVID-19 history, including comorbidities (yes/no) and, if they had contracted COVID-19, the severity of the infection (mild, moderate, or severe). COVID-19 vaccination status was also recorded, with response options indicating whether they were vaccinated (yes/no) and, if vaccinated, which vaccine they received (Pfizer, Moderna, AstraZeneca, and/or Johnson & Johnson vaccine).

To asses vaccine acceptance and hesitancy, the questionnaire included predefined response options. For vaccine acceptance, participants could select one or more of the following reasons: "recommended by health authorities," "individual health and the well-being of others," "due to external constraints," "to facilitate travel," "the beneficial effects of vaccination outweigh its side effects," "other” (with an option to specify). For vaccine hesitancy, the response options included “I don't believe in vaccination," “mass-media influence," "fear of side effects," "religious reasons," and "others."

The participants were also asked about their willingness to receive a third COVID-19 vaccine dose and to provide reasons for either acceptance or refusal.

The questionnaire included items to evaluate agreement or disagreement with influenza, pneumococcal, and meningococcal vaccinations (Yes/No). Finally, the participants were asked about their vaccination history, with responses categorized as Yes or No. Considering the complexity and importance of non-COVID vaccination among patients with diabetes, these data will be analyzed in a separate study, which will more thoroughly address behaviors related to these vaccines (influenza, pneumococcal, and meningococcal). This study will provide a deeper understanding of vaccine acceptance within this patient group, focusing on non-COVID preventive vaccination.

Statistical analysis

Data were analyzed using IBM SPSS Statistics for Windows, Version 23.0 (released 2014, IBM Corp., Armonk, NY). The sample size was estimated using the prevalence of patients willing to respond to the questionnaire regarding vaccination against COVID-19 set at 10%, a 95% confidence interval, and a relative precision of 5%. The minimum sample size was calculated using online software, Open Epi, and estimated at 135 (10%). The difference between groups that accepted or refused vaccination was examined using the Chi-square test, p-value < 0.05, and was considered significant. If the results were statistically significant, we calculated the odds ratio (OR) with a confidence interval of 95%. Where needed, we applied the Yates correction to the Chi-square.

Ethical consideration

The protocol was approved by the Ethics Review Board (No. 206, approval date: October 21, 2024) of the University of Medicine and Pharmacy “Iuliu Hațieganu” from Cluj-Napoca. At the first interaction with the HCWs, all study participants were informed about the use and anonymization of the data. Written informed consent was obtained from each participant in the study before the questionnaire was administered.

No form of incentive was provided to the subjects of the survey.

## Results

One-hundred eighty-nine out of 200 diabetic patients participated in the study; among them, 186 had type 2 diabetes and three had type 1 diabetes. The acceptance rate for completing the questionnaire was 94.5%. From the total number of participants, 96 (50.8%) were female and 93 (49.2%) males. Only persons who were 18 years old were included, and no minors were involved in this study. The COVID-19 vaccination rate was 91.5%. Most patients were aged 51 to 70 (116; 61.4%). Most of them were married (155; 82%), living with persons older than 65 years (47; 24.9%), and having one or more chronic diseases (117; 61.9%). Cardiovascular diseases were the most common comorbidities (94; 49.7%). Regarding the employment status, 109 (57.7%) were retired. Moreover, regarding ethnicity and religion, 159 (84.1%) were Romanian, and 144 (76.2%) of the responders were Orthodox. Regarding education, 56 (29.65%) of the responders had completed high school (Table 1).

**Table 1:** Demographic characteristics of patients with diabetes mellitus. p < 0.05 was considered statistically significant; chi-square

Variable		Total – n (%)	COVID-19 vaccinated – n (%)	COVID-19 not vaccinated – n (%)	χ²	p
Age	18-30	1 (0.5)	0 (0)	1 (100.0)	17.9	0.001
	31-50	12 (6.4)	9 (5.2)	3 (18.8)		
	51-70	116 (61.4)	106 (61.3)	10 (62.5)		
	71-90	60 (31.7)	58 (33.5)	2 (12.5)		
Gender	Female	96 (50.8)	89 (51.4)	7 (43.8)	0.02	0.556
	Male	93 (49.2)	84 (48.6)	9 (56.3)		
Ethnicity	Romanian	159 (84.1)	148 (85.5)	11 (68.8)	7.31	0.026
	Hungarian	25 (13.2)	22 (12,7)	3 (18.8)		
	Other	5 (2.6)	3 (1,7)	2 (12.5)		
Marital status	Married	155 (82)	142 (82.1)	13 (81.3)	19.68	< 0.001
	Unmarried	3 (1.6)	1 (0.6)	2 (12.5)		
	Divorced	2 (1.1)	1 (0.6)	1 (6.3)		
	Widow	29 (15.3)	29 (16.8)	0 (0.0)		
Living with	Children	70 (37)	60 (34.7)	10 (62.5)	6.71	0.152
	Persons >65 years old	47 (24.9)	46 (26.6)	1 (6.3)		
	Persons with chronic diseases	32 (16.9)	29 (16.8)	3 (18.8)		
	Alone	31 (16,4)	30 (17.3)	1 (6.3)		
	Didn't answer	9 (4.8)	8 (4.6)	1 (6.3)		
Occupation	Employed	55 (29.1)	46 (26.6)	9 (56.3)	8.71	0.033
	Unemployed	12 (6.3)	10 (5.8)	2 (12.5)		
	Retired	109 (57.7)	105 (60.7)	4 (25)		
	Didn't answer	13 (6.9)	12 (6.9)	1 (6.3)		
Religion	Orthodox	144 (76.2)	137 (79.2)	7 (43.8)	10.38	0.006
	Catholic	12 (6.3)	10 (5.8)	2 (43.8)		
	Other	33 (17.5)	26 (15)	7 (43.8)		
Education	Primary school	61 (32.3)	4 (5.2)	57 (55.8)	10.51	0.023
	High school	56 (29.6)	53 (30.6)	3 (18.8)		
	Higher education	28 (14.8)	21 (12.1)	7 (43.8)		
	Trade school	42 (22.2)	40 (23.1)	2 (12.5)		
	Didn't answer	2 (1.1)	2 (1.2)	0 (0.0)		

A strong significance level was found for marital status (p < 0.001), age (p = 0.001), and religion (p = 0.006). Moreover, we found a significant p-value for ethnicity (p = 0.026), occupation (p = 0.033), and education (p = 0.023). Married patients aged between 51 and 71 years old and having an orthodox religion have the highest vaccine acceptance. Retirement and high school significantly influence vaccine acceptance (Table 1).

Most patients had underlying health conditions, with 117 (61.9%) experiencing comorbidities. The proportion of comorbidities was similar between vaccinated and unvaccinated individuals. The most common comorbidities were cardiovascular diseases and obesity. Nearly half of the participants (94; 49.7%) had cardiovascular conditions, with similar prevalence between vaccinated and unvaccinated individuals. A higher percentage of unvaccinated individuals had obesity compared to those vaccinated, although this difference was not statistically significant. Only a smaller proportion of participants reported allergies or other conditions, all vaccinated. The findings indicate no significant differences in comorbidities between vaccinated and unvaccinated groups, suggesting that underlying health conditions did not significantly influence vaccination decisions (Table 2).

**Table 2:** Distribution of comorbidities in patients with diabetes mellitus. p < 0.05 was considered statistically significant; chi-square

Variable		Total – n (%)	COVID-19 vaccinated – n (%)	COVID-19 not vaccinated – n (%)	χ²	p
Comorbidities	Yes	117 (61.9)	108 (62.4)	9 (56.3)	0.24	0.887
	No	91 (27.5)	47 (27.2)	5 (31.3)		
	Didn´t answer	20 (10.6)	18 (10.4)	2 (12.5)		
	Obesity	37 (19.6)	32 (18.5)	5 (31.3)	0.56	0.419
	Cardiovascular	94 (49.7)	87 (50.3)	7 (43.8)	0.55	0.878
	Allergies	6 (3.2)	6 (3.5)	0 (0.0)	0.61	0.734
	Other	5 (2.6)	5 (2.9)	0 (0.0)	0.51	0.769

A significant proportion of the study population, 161 (85.2%), reported contracting COVID-19, with 28 (14.8%) remaining uninfected. This demonstrates that most participants in this cohort were affected by the virus, underlining the widespread nature of COVID-19 within the studied population. Most patients experienced mild or moderate forms of the disease, with a relatively lower incidence of severe cases within this group. This predominantly mild-to-moderate distribution of illness severity could be attributed to various factors, including the high vaccination rate observed among our patients. Regarding underlying health conditions, most patients are known to have comorbidities that could have played a role in shaping these outcomes (Table 3).

**Table 3:** Diabetic patients' COVID-19 status.

Variable		Total – n (%)
COVID-19 infection	Yes	161 (85.2)
	No	28 (14.8)
COVID-19 form	Mild	87 (54)
	Moderate	73 (45.3)
	Severe	1 (0.6)

The highest frequency of COVID-19-vaccinated participants in the study was observed in patients with type 2 diabetes mellitus (98.4%). Moreover, regarding diabetes treatment, most patients who were COVID-19-vaccinated take oral antidiabetics (162; 85.7%). Given that most patients fall within the 51-70 age range and still have a high rate of oral antidiabetic treatment, it suggests that they adhere to medical recommendations for diabetes management. This adherence likely contributes to increased protection against severe forms of COVID-19 (Table 4).

**Table 4:** Type of diabetes and the antidiabetic treatment administered in COVID-19 vaccinated patients.

Variable		Total – n (%)
Diabetes	Type 1	3 (1.6)
	Type 2	186 (98.4)
Treatment	Insulin	33 (17.5)
	Oral antidiabetics	162 (85.7)

The most commonly administered vaccine was Pfizer, accounting for 143 (82.7%) of all vaccines administered, followed by Moderna 19 (11%). AstraZeneca and Johnson & Johnson vaccines were less frequently administered. However, the relatively lower uptake of AstraZeneca and Johnson & Johnson vaccines might point to vaccine hesitancy regarding their safety profiles. Factors like age, underlying health conditions, and prior knowledge of vaccine safety play key roles in influencing vaccine choices (Table 5).

**Table 5:** Types of COVID-19 vaccine administered.

Variable		Total – n (%)
Vaccine	Pfizer	143 (82.7)
	Moderna	19 (11)
	Astra-Zeneca	6 (3.5)
	Johnson & Johnson	5 (2.9)

Moreover, patients were asked about the reasons for not getting vaccinated against COVID-19. The principal reason is the fear of side effects (7; 43.8%). Personal perception seems to be implicated in vaccine hesitancy for the third dose (22; 20.6%). From the total number of vaccinated patients, a substantial part, 82 (76.6%), did not wish to respond as to whether they agreed or disagreed with the administration of the third dose of the COVID-19 shot (Table 6).

**Table 6:** COVID-19 vaccination hesitancy.

Variable		Total – n (%)
Vaccine hesitancy	Fear of side effects	7 (43.8)
	Mass media influence	2 (12.5)
	Personal perception	2 (12.5)
	Other	3 (18.8)
	No answer	2 (12.5)
Third dose	No	107 (61.8)
Third dose vaccine hesitancy	Fear of side effects	2 (1.9)
	Personal perception	22 (20.6)
	Lack of trust	1 (0.9)
	Recommendations from health authorities	1 (1.5)
	No answer	82 (76.6)

The analyzed data regarding vaccine acceptance indicate that the main reasons patients with diabetes chose to get vaccinated were individual health and the well-being of others (63%), followed by recommendations from health authorities (16.8%) and the belief that the benefits of vaccination outweigh the side effects (15%). Of the 173 COVID-19 vaccinated patients, 38.2% would get vaccinated with the third dose. Moreover, 84.8% of them said that the main reason they would get vaccinated with the third dose is individual health and the well-being of others. Individuals with chronic health conditions, such as diabetes, tend to exhibit higher vaccine acceptance due to their heightened risk of severe illness if infected with COVID-19. Our data showed a high vaccination rate among diabetes patients (Table 7).

**Table 7:** COVID-19 vaccination acceptance.

Variable		Total – n (%)
Vaccine acceptance	Individual health and the well-being of others	109 (63)
	Recommendations from health authorities	29 (16.8)
	The beneficial effects of vaccination outweigh its side effects.	26 (15)
	No answer	9 (5.2)
Third dose	Yes	66 (38.2)
Third dose vaccine acceptance	Individual health and the well-being of others	56 (84.8)
	Recommendations from health authorities	1 (1.5)
	No answer	9 (13.6)

Age (OR = 5.57), university degrees (OR = 5.03), and religion (OR = 4.25) are significant factors that are more likely to influence COVID-19 vaccination (Table 8). 

**Table 8:** Odds ratio of determinants of COVID-19 vaccination. Odds ratio; p < 0.05 was considered statistically significant; *Yates corrected Chi-square / Chi-square

Variable	OR	95%CI	χ²	p
Age	5.57	1.509–20.567	5.48	0.019*
Religion	4.25	1.531–11.795	7.06	0.008*
University degree	5.033	1.729–14.649	8.12	0.004*
Work active	3.082	1.122–8.464	3.96	0.047*
Diabetes complications	3.968	1.243–12.662	6.125	0.013

## Discussion

The present study aimed to asses the determinants of COVID-19 vaccine acceptance in patients with diabetes regarding personal opinion and sociodemographic and medical characteristics and highlight some of these factors that are believed to determine the acceptance of COVID-19 vaccination.

The study identified several factors associated with COVID-19 vaccine acceptance in patients with diabetes mellitus, such as marital status, age, ethnicity, occupation, religion, and the reasons that influenced vaccine acceptance or hesitancy. In terms of comorbidity status, participants with diabetes or other chronic conditions, such as cardiovascular diseases, may exhibit differing susceptibility to COVID-19 severity. The distribution of administered vaccines may reflect national vaccination policies, vaccine availability, or public preferences regarding specific manufacturers. The data highlight the predominant reliance on mRNA vaccines (Pfizer and Moderna) in this cohort, which aligns with broader global trends favoring these vaccine types due to their high efficacy and widespread availability.

The main reason for COVID-19 vaccine acceptance identified in our study is related to personal health and the health of those around us. This was also the main reason for accepting the third vaccine dose. To a lesser extent, it is followed by recommendations from health authorities and the belief that the benefits of vaccination outweigh the adverse effects. Regarding COVID-19 vaccine hesitancy, the main reason for not getting vaccinated was the fear of side effects. Personal perception seems to be implicated in vaccine hesitancy for the third dose.

Several studies showed that marital status contributes positively to vaccine acceptance among married persons compared with unmarried, divorced, or widows. An American national study about marital differences regarding COVID-19 vaccine acceptance has shown that married persons are more likely to receive more than one dose of the COVID-19 vaccine [20]. Nowadays, our society tends to have more unmarried people. Social media platforms can influence our perception of ourselves, lowering our self-esteem and confidence and creating false disadvantages between people, leading to loneliness [21]​​​​. The main reasons people are single were poor flirting skills, freedom, fear of getting hurt, having different priorities, and being too picky [22]​​​​.

When considering religion, things can be varied; religion seems to be a debated issue regarding vaccination. In our study, orthodox religion had a positive impact on COVID-19 vaccination. A cross-sectional study showed vaccine hesitancy among religious groups. For White Christians, the most endorsed reasons were related to the fact that the vaccine was developed too fast or that they do not have enough information about it, concerns about side effects, and lack of trust in vaccines [22]​​​​.

The vast majority of the participants in this study were in the 51-70 age range, which has a favorable influence on COVID-19 vaccine acceptance. An Italian survey performed on 1,176 subjects affected by type 2 diabetes showed that older patients (aged >61 years) are more likely to accept the COVID-19 vaccine [23]​​​​.

One of the key negative predictors for COVID-19 vaccination, as identified in another cross-sectional survey conducted in rural Ghana, was being Christian [24]. Another research study conducted in 2021 with nearly 2,000 American participants revealed that Christian nationalism was the strongest factor influencing COVID-19 vaccine hesitancy [25].

Moreover, in our study, the values for the ethnicity variable categorize it as a reason for COVID-19 vaccine acceptance. In a population-based cross-sectional study from Nigeria among the Christian population, ethnicity was a positive determinant for vaccination [19].

Occupation can influence vaccine acceptance in different ways. Our findings demonstrate that retired diabetic patients seem to have better vaccination acceptance. Among 1,539 participants of a study from Indonesia, being retired was associated with a low vaccination acceptance [26].

Factors such as a higher level of education, good knowledge, a favorable attitude, a previous history of COVID-19 infection, being male, and having chronic diseases were identified as predictors of COVID-19 vaccine acceptance [27]. A study from Romania that assessed the influence of knowledge toward vaccination among medical students at the beginning and end of an Immunology course showed a positive shift in attitude toward current and future vaccines correlated with improvement of knowledge [28].

A cross-sectional survey showed that patients with severe comorbidities were more likely to refuse COVID-19 vaccination. Vaccine hesitancy was reported by 19.7% of individuals with obesity, 18.0% of those diagnosed with hypertension, and 19.0% of individuals with type 2 diabetes, compared to respondents without these comorbidities [29]. A South Korean study evaluating the safety and adverse effects of COVID-19 vaccines, including Pfizer, Moderna, AstraZeneca, and Janssen, showed that, given the increased risk of severe complications or death in individuals with comorbidities when infected with COVID-19, the benefits of vaccination outweigh the potential risks. The interim guidelines for COVID-19 vaccines from Pfizer, Moderna, and AstraZeneca recommended that all three vaccines be administered to individuals with high-risk comorbidities [30].

During the pandemic, preventive measures like vaccination were essential to limit the impact of COVID-19, particularly for vulnerable populations like those with chronic conditions, such as diabetes. The introduction of Pfizer's vaccine played an important role in reducing severe cases and hospitalizations, especially among high-risk groups [31].

A study using a model to identify factors influencing vaccine hesitancy revealed that the lowest acceptance rates were in Burkina Faso (66.5%) and Pakistan (66.5%). The principal determinant for vaccine acceptance was the interest in personal protection against COVID-19; side effects were implicated in vaccine hesitancy. Moreover, HCWs were the most trusted source of information regarding vaccine acceptance. An online survey from the U.S. found that unemployed participants had lower COVID-19 vaccine acceptance than those employed or retired. [32]. To overcome the obstacles of vaccine hesitancy, awareness initiatives must be strengthened to increase the possibility of acceptance among specific populations, such as diabetic patients. It is vital to stimulate the perception of risk and severity among these particular groups of patients. 

The present study has several limitations that need to be acknowledged. First, we had a small sample size, and the study population was derived from only two medical centers. Second, the limitations of the cross-sectional study design and the use of a questionnaire may have influenced the findings. Using a cross-sectional study design, we may have encountered susceptibility to selection, information bias, and limited generalizability. Using a questionnaire can lead to misinterpretation of questions, limited depth of responses, potential for incomplete or inaccurate data, and a low response rate. Since the sample was drawn from specific medical centers rather than randomly from the general diabetic population, findings may not be representative of all diabetic patients Potential confounders, such as demographic characteristics (age, education, and socioeconomic status), medical history (severity of diabetes, presence of comorbidities, history of COVID-19 infection, and current treatment), psychological factors (trust in healthcare, personal motivation, and fear of side effects), social and environmental variables (family, individuals in the social circle, vaccination policies, and recommendations from authorities) can influence a patient's decision to accept or refuse the vaccine.

However, our findings provide important and useful information on potential strategies to optimize vaccine uptake among these high-risk populations. According to our knowledge, the present study is the first research on patients with diabetes from Cluj-Napoca, one of the riskiest categories of complications due to not being vaccinated. COVID-19 remains a public health problem due to its contagiousness rate, new strains that can resist the vaccine, seasonality, and vaccination hesitancy.

The interest in this topic is growing exponentially, and considerable efforts have been made to understand the missing piece about vaccine hesitancy and promote vaccination as a part of a healthy lifestyle [33]. Social media is undoubtedly one of the most widely used methods for socializing, communicating, and sharing information. These platforms could promote information about vaccination and sustain public health by creating quality content [34]. Among the most important ways to promote a healthy lifestyle to prevent any disease or disability is good communication based on the medical information of the general population.

A strong education regarding preventive medicine starts from a young age, and open professional communication is the key to understanding and accepting medical care, including vaccination. The health system plays an important role in promoting and implementing vaccination. Each HCW, no matter the department where they work, should talk with their patients about the importance of immunization. These days, people use the internet as a source of medical information, with the risk of endangering their health or that of others if they get it from the wrong sources. It would be great if preventive medicine and learning how to find a good source of information were integrated into the educational system.

## Conclusions

 Knowing the influence of age, partnership, occupation, ethnicity, level of education, and religion on COVID-19 vaccine acceptance, we can adjust and enhance vaccination campaign strategies. In addition, based on the research, we can organize scales of supplying vaccine-related information according to the vaccination schedule. As observed in the study, perception levels may change as doses are administered.

This study offers essential insights for enhancing the health and well-being of patients with diabetes mellitus. It also has significant socioeconomic implications. By understanding and preventing certain situations that may escalate a public health issue, we can reduce material costs and the strain on human resources.

Because vaccination is a controversial topic and is evolving, there will always be gaps to fill, and any future study can bring valuable information.

## Appendices

Appendix A: Vaccination Acceptance Questionnaire

**Table 9:** English and Romanian version of the Vaccination Acceptance Questionnaire Legend: The English and Romanian version of the Vaccination Acceptance Questionnaire. Credits: [19]

Question Number (English)	Question Number (Romanian)	Options (English)	Options (Romanian)
1. Age	1.Varsta dumneavoastra	a) 18-30 years, b) 31-50 years, c) 51-70 years, d) 71-90 years	a) 18-30 ani, b) 31-50 ani, c) 51-70 ani, d) 71-90 ani
2. Gender	2. Sex	a) female, b) male	a) f, b) m
3. Ethnicity	3. Etnie	a) Romanian, b) Hungarian, c) Roma, d) Other	a) Romana, b) Maghiara, c) Rroma, d) Alta
4. Marital status	4. Status marital	a) married, b) single, c) divorced, d) widowed	a) casatorit/a, b) necasatorit/a, c) divortat/a, d) vaduv/a
5. Do you live with	5. Locuiti cu	a) children, b) people over 65 years, c) people with chronic diseases, d) alone	a) copii, b) persoane >65 ani, c) persoane cu boli cronice, d) singur/a
6. Occupation	6. Ocupatie	a) retired, b) unemployed, c) high-risk job (exposure to pathogens such as healthcare), d) medium risk (pharmacist, supermarket, etc.), e) low risk (IT, office staff, etc.)	a) pensionar, b) nu lucrez, c) munca la risc mare (expunere la diversi agenti patogeni-ex.sistem de sanatate), d) risc mediu (farmacist, supermarket etc), e) risc mic (IT, personal de birou etc)
7. Religion	7. Religie	a) Orthodox Christian, b) Catholic, c) Other	a) Crestin-Ortodoxa, b) Catolica, c) Alta
8. Education	8. Studii	a) primary school, b) high school, c) vocational school, d) higher education	a) studii primare, b) liceul, c)scoala profesionala, d)studii superioare
9. What type of diabetes do you have?	9.Ce tip de diabet zaharat aveti?	a) type 1 diabetes, b) type 2 diabetes, c) gestational diabetes	a) diabet zaharat tip 1, b)diabet zaharat tip 2, c)diabet gestational
10. Are you on insulin treatment?	10. Sunteti sub tratament cu insulina?	a) yes, b) no	a) da, b) nu
11. If YES, how many doses of insulin do you take?	11. Daca DA, cate doze de insulina va administration?	a) 1 dose, b) 2 doses, c) 3 doses, d) 4 doses	a) 1 doza, b) 2 doze, c) 3 doze, d) 4 doze
12. Do you know the name of the insulin you are using?	12.Cunoasteti denumirea insulinei administrate?	a) If yes, please name it, b) no	a) daca DA, care este, b) Nu
13. Are you on oral antidiabetic treatment?	13.Urmati tratament cu antidiabetice orale?	a) yes, b) no	a) da, b) nu
14. Do you know the name of the oral antidiabetics you are taking?	14. Cunoasteti denumirea antidiabeticelor orale pe care le luati?	a) If yes, please name the medication, b) no	a) daca DA, care sunt, b) nu
15. Do you have any diabetes complications?	15. Complicatii ale diabetului zaharat?	a) ocular, b) renal, c) cardiovascular, d) diabetic foot, e) dermatological, f) none	a) oculare, b) renale, c) cardiovasculare, d) picior diabetic, e) dermatologice, f) fara
16. Do you have other diseases?	16. Aveti alte boli?	a) obesity, b) cardiovascular diseases, c) allergies, d) other (e.g., neoplasms, liver, kidney, eye diseases)	a) obezitate, b) boli cardiovasculare, c) alergii, d) altele, cuma ar fi: neoplazii, boli de ficat, rinichi, ochi.
17. Have you had COVID-19?	17. Ati facut COVID-19?	a) yes, b) no	a) da, b) nu
18. If YES, what was the severity?	18. Daca ati facut COVID-19, ce forma a fost?	a) mild, b) moderate, c) severe	a) usoara, b) medie, c) severa
19. Have you been vaccinated against COVID-19?	19. V-ati vaccinate?	a) yes, b) no	a) da, b) nu
20. If YES, which vaccine did you receive?	20. Daca DA, cu ce vaccin?	a) Pfizer, b) Moderna, c) AstraZeneca, d) Johnson & Johnson	a) Pfizer, b) Moderna, c) AstraZeneca, d) Johnson & Johnson
21. If YES, what was your reason for vaccination?	21. Daca DA, de ce v-ati vaccinat?	a) recommended by health authorities, b) for my health and the protection of others, c) under pressure, d) to travel, e) because the benefits of vaccination outweigh the adverse effects, f) other, please specify:	b) pentru sanatatea mea si protectia celor din jur, c)din constrangere, d) pentru a calatori, e) pentru ca efectele befefice ale vaccinarii depasesc efectele adverse ale acesteia, f) altele, specificati
22. If NO, what was your reason for not getting vaccinated?	22. Daca NU, de ce nu v-ati vaccinat?	a) I don't believe in vaccination, b) media influence, c) fear of side effects, d) for religious reasons, e) other, please specify:	a) nu cred in vaccinare, b) influenta mass-media, c) teama de efecte adverse, d) din motive religioase, e) altele, specificati:
23. Would you get a third dose?	23. V-ati mai vaccina cu a 3-a doza?	a) yes, b) no	a) da, b) nu
24. If YES, why would you get the third dose?	24. Daca DA, de ce v-ati vaccina cu doza 3?	-	-
25. If NO, why would you not get the third dose?	25. Daca NU, de ce nu v-ati vaccina cu doza 3?	-	-
26. According to health studies, vaccination is recommended for diabetes patients:	26. Conform studiilor pentru sanatate, la pacientii diabetici se recomanda:	-	-
a) Flu vaccination - Do you agree with this vaccination?	a) vaccinarea antigripala. Sunteti de acord cu aceasta vaccinare?	a) yes, b) no	a) da, b) nu
b) Pneumococcal vaccination – Do you agree with this vaccination?	b) vaccinarea pneumococica. Sunteti de acord cu aceasta vaccinare?	a) yes, b) no	a) da, b) nu
27. Vaccination history	27. Istoric de vaccinare	a) I was vaccinated in childhood (measles/mumps/rubella; hepatitis B; tuberculosis; polio; diphteria/tetanus/pertussis; Haemophilus influenza; HPV)	a) Am fost vaccinat/a in copilarie(rujeola/oreion/rubeola; anti hepatita, b) anti tuberculoza; anti poliomielita; difteri/tetanos/tuse convulsiva; anti hemophilus influenzae; anti HPV)
		b) I was not vaccinated in childhood.	b) Nu am fost vaccinat/a in copilarie.
